# Study of the biological potential of *in vitro *extracts for *Zeyheria tuberculosa *(Bignoniaceae)

**DOI:** 10.1186/1753-6561-8-S4-P13

**Published:** 2014-10-01

**Authors:** Isabelle Souza de Mélo Silva, Raíssa Fernanda Evangelista Pires dos Santo, Naélia Monique Moreira Brito Silva, Andressa Leticia Lopes da Silva, Ingridy Viana Lucena, Andriele Mendonça Barbosa, Klebson Silva Santos, Thiane de Vasconcellos Costa Melo, Genilson Sarmento Lins Júnior, Francine Ferreira Padilha, Patricia de Albuquerque Sarmento, Maria Lysete de Assis Bastos

**Affiliations:** 1 Instituto de Tecnologia e Pesquisa, Universidade Tiradentes, Av. Murilo Dantas, 300, Farolândia, Aracaju, Sergipe, CEP 49032-490, Brazil; 2 Laboratório de Pesquisa em Tratamento de Feridas, Universidade Federal de Alagoas, Campus A. C. Simões, Av. Lourival Melo Mota, s/n, Cidade Universitária, Maceió, Alagoas, CEP 57072-900, Brazil

## Background

The indiscriminate use of antibiotics, associated with the emergence of microorganisms human resistant pathogens to major classes of antibiotics, has caused many clinical problems in the treatment of infectious diseases. Plants used in order to medicinal purpose for treatment, healing and/or prevention is one of the oldest forms of medicinal practice of humanity [1]. Biomonitored studies are developed and refined, relating popular knowledge to realization of bioassays that confirm the therapeutic efficacy and the low toxicity, as the plants with efficacy proven for a particular biological activity are possible raw materials for natural and/or synthetic medicines [2]. The aim of this study was to evaluate the antibacterial, cytotoxic and antioxidant potential *in vitro *of extracts of a native plant from Alagoas, the *Zeyheria tuberculosa*, known as Ipê Felpudo used in Brazilian folk medicine for the treatment of cancer and skin diseases [3].

## Methods

*In vitro *experimental research, was realized in the Laboratory of Wound Care at Federal University of Alagoas. Four fractions were tested in different parts (leaves, stems) of *Zeyheria tuberculosa*, extracts XL_1_. XL_2_, XL_3 _and XS_1_. Antimicrobial activity was determined by microbial sensitivity tests, the method of well diffusion, plates-holes diffusion assay and broth microdilution method for determination of minimum inhibitory concentration (MIC). The bacterial inhibition percentage of disk diffusion test was calculated by dividing the mean of the sample inhibition, for hundred times by the mean of inhibition halos for the positive control[4]. Were used eight bacterial strains, among them Gram-positive and Gram-negative bacteria, distributed by *American Type Cell Cellection*. The evaluation of the samples toxicity was obtained through toxicity test with *Brine Shrimp*. The evaluation of the free radical sequester ability was performed according to the free radical sequester activity of the synthetic 2,2-Diphenyl-1-picrylhydrazyl (DPPH).

## Results and conclusions

All tested samples showed moderately active against the strain of *Sthapylococcus aureus *(ATCC 25923) using the agar diffusion method by the well technique with percentage of inhibition >25% and <75%. The XS_1 _extract showed the most significant antimicrobial activity in MIC, inhibiting the strain growth of *S. aureus *with 1000 until 125 μg mL^-1 ^concentration. Identified the absence of toxicity in all samples, as the mortality percentage rate was ≤30% (CL_50 _≥ 1000 µg mL^-1^). In the antioxidant test, all samples were considered inactive with CL_50_> 200 mg/mL. Phytochemical studies previously performed with *Z. tuberculosa *showed the presence of isolated flavonoids justifying the antimicrobial activity found [3]. These results represent the primary indications security plant species for performing *in-vivo *bioassays with perspective in the infection control.
